# Co‐Delivery of aPD‐L1 and CD73 Inhibitor Using Calcium Phosphate Nanoparticles for Enhanced Melanoma Immunotherapy with Reduced Toxicity

**DOI:** 10.1002/advs.202410545

**Published:** 2024-12-24

**Authors:** Peng Liu, Jia Guo, Zuozhong Xie, Yusheng Pan, Benliang Wei, Ying Peng, Shuo Hu, Jinsong Ding, Xiang Chen, Juan Su, Hong Liu, Wenhu Zhou

**Affiliations:** ^1^ Xiangya School of Pharmaceutical Sciences Central South University Changsha Hunan 410013 China; ^2^ Department of Nuclear Medicine Xiangya Hospital Central South University Changsha Hunan 410008 China; ^3^ Key Laboratory of Biological Nanotechnology Changsha Hunan 410008 China; ^4^ Department of Dermatology, Xiangya Hospital Central South University Changsha Hunan 410008 China; ^5^ National Engineering Research Center of Personalized Diagnostic and Therapeutic Technology Changsha Hunan 410008 China; ^6^ Hunan Key Laboratory of Skin Cancer and Psoriasis Changsha Hunan 410008 China; ^7^ Department of Otorhinolaryngology Head and Neck Surgery, The Second Xiangya Hospital Central South University Changsha Hunan 410011 China; ^8^ Big Data Institute Central South University Changsha Hunan 410083 China

**Keywords:** biomineralization, drug delivery, immune checkpoint inhibitors, melanoma, synergistic therapy, tumor targeting

## Abstract

Melanoma, a malignant skin tumor, presents significant treatment challenges, particularly in unresectable and metastatic cases. While immune checkpoint inhibitors (ICIs) targeting PD‐1/PD‐L1 have brought new hope, their efficacy is limited by low response rates and significant immune‐mediated adverse events (irAEs). Through multi‐omics data analysis, it is discovered that the spatial co‐localization of CD73 and PD‐L1 in melanoma correlates with improved progression‐free survival (PFS), suggesting a synergistic potential of their inhibitors. Building on these insights, a novel therapeutic strategy using calcium phosphate (CaP) nanoparticles is developed for the co‐delivery of aPD‐L1 and APCP, a CD73 inhibitor. These nanoparticles, constructed via a biomineralization method, exhibit high drug‐loading capacity and pH‐responsive drug release. Compared to free aPD‐L1, the CaP‐delivered aPD‐L1 effectively avoids systemic side effects while significantly enhancing anti‐tumor efficacy, surpassing even a 20‐fold dose of free aPD‐L1. Furthermore, the co‐delivery of aPD‐L1 and APCP via CaP nanoparticles demonstrates a synergistic anti‐tumor effect, with substantial immune activation and prevention of tumor recurrence through immune memory effects. These findings suggest that the co‐delivery of aPD‐L1 and APCP using CaP nanoparticles is a promising approach for improving melanoma immunotherapy, achieving enhanced efficacy and reduced toxicity.

## Graphical abstract

This study discovers that the spatial co‐localization of CD73 and PD‐L1 in melanoma correlates with improved progression‐free survival. Building on these insights, this study develops a novel therapeutic strategy using calcium phosphate nanoparticles for the co‐delivery of APCP and aPD‐L1, achieving synergistic melanoma immunotherapy and reduced irAEs.

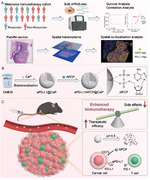

## 1 Introduction

Melanoma is a malignant skin tumor with a poor prognosis.^[^ ^1^ ^]^ While early‐stage melanoma can often be effectively treated through surgical resection, treatment options for unresectable melanoma or patients with distant metastasis are limited and generally unsatisfactory. Recently, immunotherapy, particularly immune checkpoint inhibitors (ICIs) targeting PD‐1/PD‐L1, has emerged as a promising approach in melanoma treatment.^[^ ^2^ ^]^ In 2014, Nivolumab became the first PD‐1 inhibitor approved by the FDA for the treatment of melanoma. Despite the success of ICIs, only a subset of patients benefits from these treatments, and the overall response rate remains relatively low.^[^ ^3^ ^]^ Furthermore, compared to chemotherapy or targeted therapies, ICIs are associated with an increased risk of organ‐specific immune‐mediated adverse events (irAEs).^[^ ^4^ ^]^ Due to the expression of PD‐L1 in normal tissues, intravenous administration of aPD‐L1 may result in its binding to non‐tumor tissues, leading to reduced concentrations of aPD‐L1 at the tumor site and diminished efficacy.^[^ ^5^ ^]^ Additionally, this can cause non‐specific immune activation and on‐target off‐tumor irAEs, including elevated transaminase levels, pneumonia, and nephritis, which can necessitate treatment interruption or even lead to fatal outcomes.^[^ ^6^ ^]^ Therefore, it is crucial to enhance the therapeutic efficacy and safety of ICIs.

Recent clinical trial results indicate that combining PD‐1/PD‐L1 antibodies with other immune checkpoint inhibitors significantly improves response rates (RR), progression‐free survival (PFS), and overall survival (OS) compared to PD‐1/PD‐L1 monotherapy.^[^ ^7^ ^]^ CD73 inhibitors, for example, have shown synergistic effects with PD‐(L)1 inhibitors in certain tumors and have demonstrated promising results in patients with advanced solid tumors. CD73, a cell surface enzyme encoded by NT5E, is widely expressed on various cell surfaces and is upregulated in the tumor microenvironment under hypoxic conditions.^[^ ^8^ ^]^ CD73, in concert with CD39, converts ATP, which has immune activation functions, into adenosine, which has immune suppressive effects.^[^ ^9^ ^]^ Adenosine downregulates immune activity by binding to A2AR and other adenosine receptors.^[^ ^10^ ^]^ It has been reported that CD73 is upregulated in melanoma tissues compared to peritumoral tissues, and its high expression is associated with tumor metastasis, reduced response to immunotherapy, and poor prognosis, including worse overall survival (OS) and disease‐free survival (DFS).^[^ ^11^ ^]^ This suggests that CD73 could be a promising therapeutic target in melanoma. Increasing evidence supports CD73 as a novel immune checkpoint. Clinical trials exploring the combination of CD73 inhibitors with aPD‐(L)1 antibodies in advanced solid malignancies are currently underway globally (NCT04148937, NCT05431270, NCT04104672).^[^ ^12^ ^]^ However, whether the combination of CD73 inhibitors with aPD‐(L)1 can enhance immunotherapy efficacy in melanoma remains to be fully explored.

**Scheme 1:**
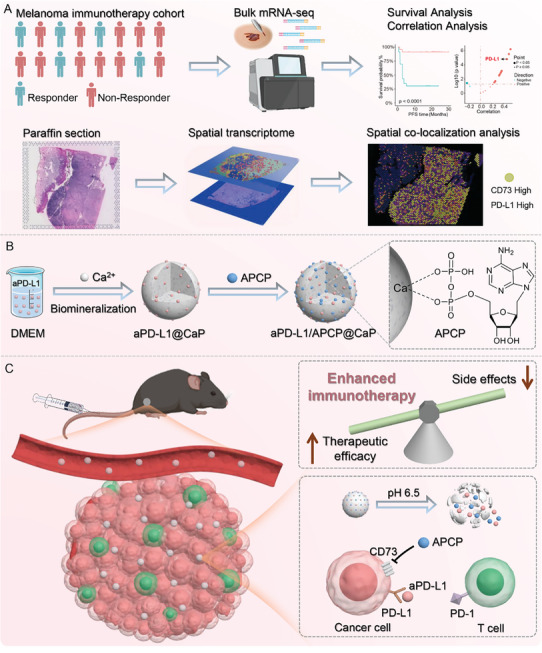
A) Illustration showing the experimental workflow for multi‐omics data analysis. B) Schematic illustration of the fabrication process of aPD‐L1/APCP@CaP and C) its in vivo performance for synergistic immunotherapy of melanoma with reduced irAEs.

In our study, Bulk transcriptome and spatial transcriptome sequencing revealed that CD73 expression is significantly positively correlated with PD‐L1 expression and shows a co‐localization trend in the spatial distribution within tumor areas (**Scheme** **1**A). This suggests that the combined inhibition of CD73 and PD‐L1 could be a potential strategy to enhance melanoma immunotherapy efficacy. Although previous study revealed that the dual immune checkpoint inhibitors contribute to the enhanced efficacy of anti‐tumor, the high rate of irAEs restricts their clinical applications.^[^ ^13^ ^]^ Based on these findings, we developed a calcium phosphate biomineralized nanomedicine for the co‐delivery of aPD‐L1 and APCP (a specific CD73 inhibitor) (Scheme 1B). Encapsulating PD‐L1 within biomineralized nanoparticles blocks aPD‐L1 binding to normal tissue cells during circulation, significantly enhances the accumulation of aPD‐L1 in tumor tissues via the enhanced permeability and retention (EPR) effect, and enables pH‐responsive degradation in the acidic tumor microenvironment to release aPD‐L1, restoring its activity and enhancing immunotherapy efficacy (Scheme 1C). This delivery strategy can avoid the irAEs associated with aPD‐L1 and significantly improve therapeutic outcomes, reducing the required dosage by twenty‐fold. Additionally, the biomineralized nanoparticles effectively adsorb APCP through calcium ion coordination, facilitating the combined delivery of aPD‐L1 and APCP. The synergistic immunotherapeutic effects and immune memory responses have been confirmed in both primary and distant metastatic tumor models. This study proposes a novel combination therapy strategy involving immune blockers for melanoma immunotherapy, and introduces new formulation approaches to enhance the efficacy and reduce the toxicity of immunotherapy.

## 2 Results and Discussion

### 2.1 Multi‐Omics Data Analysis the Potential of CD73 and PD‐L1 as Dual Target Combination in Melanoma Immunotherapy

**Figure 1:**
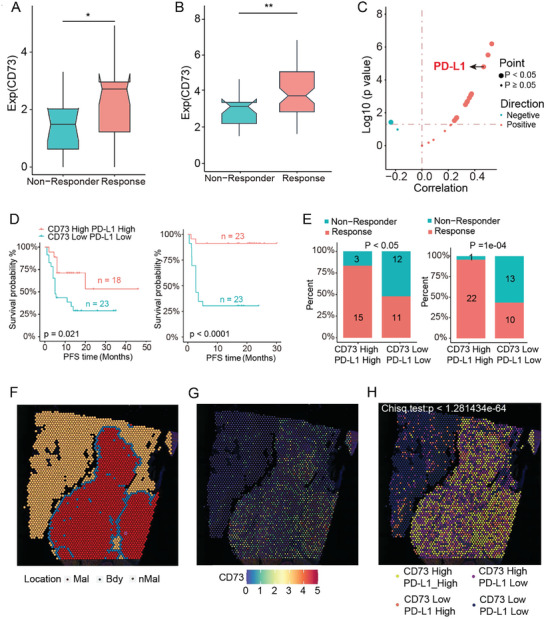
A) The Wilcoxon signed‐rank test was used to evaluate the difference in CD73 expression between response (*n* = 39) and non‐response (*n* = 29) groups in the Xiangya immunotherapy cohort. B) The Wilcoxon signed‐rank test was used to evaluate the difference in CD73 expression between response (*n* = 67) and non‐response (*n* = 21) groups in the public melanoma immunotherapy transcriptome data (PRJEB23709). C) Spearman correlation analysis of CD73 and immune checkpoint expression. D) Kaplan–Meier curves illustrating the synergistic effect on progression‐free survival (PFS) for patients stratified by CD73 and PD‐L1 expression levels in the Xiangya immunotherapy cohort (left panel) and the public melanoma immunotherapy transcriptome data (PRJEB23709) (right panel). *p*‐values were determined by the log‐rank test. E) The proportion of patients with different responses to immunotherapy in the Xiangya immunotherapy cohort (left panel) and the public melanoma immunotherapy transcriptome data (PRJEB23709) (right panel). *p*‐values were determined by Fisher's exact test. F) Four clusters identified corresponding to original histopathological annotations. G) Spatial feature map of CD73 gene expression. H) Distribution of groupings based on CD73 and PD‐L1 expression in the spatial transcriptome of melanoma. *p < 0.05, **p < 0.01.

In our analysis combining a self‐test melanoma immunotherapy cohort from Xiangya Hospital with published melanoma immunotherapy cohort data, we observed that CD73 expression was significantly higher in the response group compared to the non‐response group (**Figure** **1**A,B). This finding suggests that CD73 could serve as a potential biomarker and target for melanoma immunotherapy. Further correlation analysis between CD73 and 16 immune checkpoints revealed a significant positive correlation between CD73 and PD‐L1 expression (Figure 1C), indicating a possible synergistic effect between CD73 and PD‐L1. To explore this hypothesis, patients were divided into two groups based on CD73 and PD‐L1 expression levels: high CD73&PD‐L1 expression group and low CD73&PD‐L1 expression group. In both datasets, the high expression group of CD73&PD‐L1 was associated with improved progression‐free survival (PFS) (Figure 1D), and a higher proportion of patients in the response group compared to the low expression group of CD73&PD‐L1 (Figure 1E).

Additionally, using transcriptome data from melanoma formalin‐fixed, paraffin‐embedded (FFPE) samples, we investigated the spatial association between CD73 and PD‐L1. The Cottrazm tool was used to annotate the transcriptome into tumor regions (Mal), boundary regions (Bdy), and normal regions (nMal) (Figure 1F). CD73 was found to be widely expressed in malignant melanocytes (Figure 1G). Interestingly, spatial co‐localization of CD73 and PD‐L1 expression in the tumor area was observed (Figure 1H). These findings suggest a synergistic effect between CD73 and PD‐L1 during immunotherapy, indicating that combined inhibition of CD73 and PD‐L1 could be a promising immunomodulatory therapeutic strategy for melanoma.

### 2.2 Construction, Characterization, and In Vivo Tumor Targeting Delivery of aPD‐L1/APCP@CaP Nanoparticles

**Figure 2:**
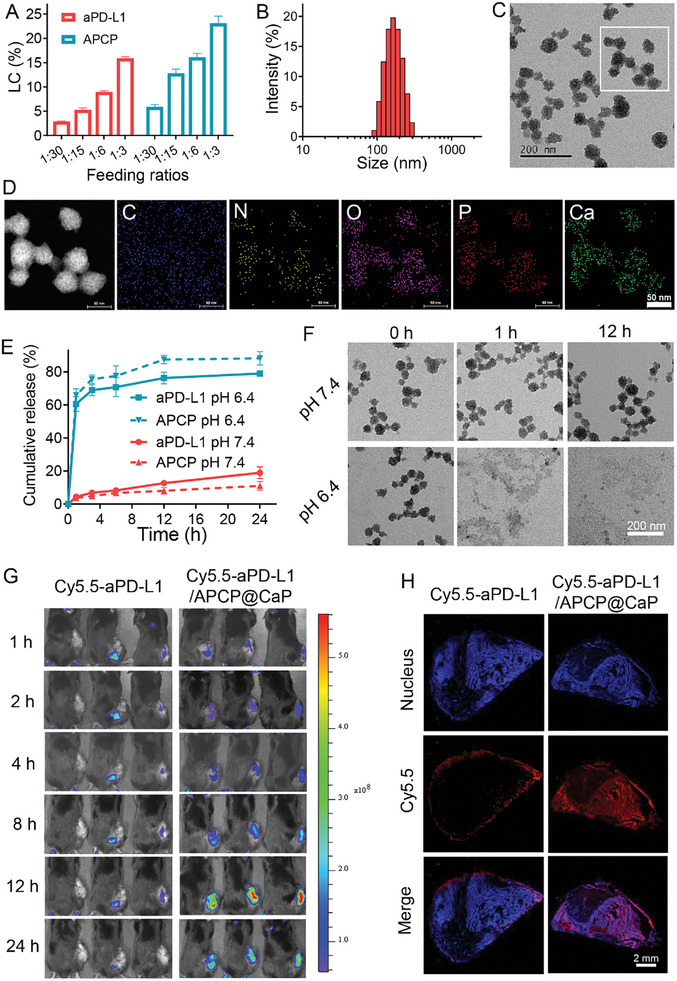
A) Drug loading capacity of aPD‐L1/APCP@CaP nanoparticles at various aPD‐L1 and APCP feeding ratios. B) DLS analysis and C) TEM image of aPD‐L1/APCP@CaP nanoparticles. D) Elemental mapping analysis showing the distribution of C, N, O, P, and Ca in aPD‐L1/APCP@CaP nanoparticles. E) In vitro release profile of aPD‐L1 and APCP from nanoparticles under neutral (pH 7.4) and acidic (pH 6.4) conditions (*n* = 3). F) TEM images depicting structural changes of aPD‐L1/APCP@CaP nanoparticles under neutral and acidic conditions over time. G) In vivo fluorescence imaging of Cy5.5‐aPD‐L1/APCP@CaP and Cy5.5‐aPD‐L1 in B16F10 tumor‐bearing mice within 24 h post‐injection. H) Distribution of Cy5.5 fluorescence in tumor tissue sections from mice treated with Cy5.5‐aPD‐L1/APCP@CaP, indicating deeper penetration compared to free Cy5.5‐aPD‐L1.

Based on the above findings, we developed a nanoparticle formulation, aPD‐L1/APCP@CaP, co‐loaded with APCP and aPD‐L1 using a biomineralization method.^[^ ^14^ ^]^ In this process, Ca^2+^ was introduced to a BSA and aPD‐L1‐containing DMEM medium. BSA and aPD‐L1 chelate Ca^2+^ to elevate the local supersaturation, which serves as nucleation sites for in situ biomineralization by reacting with the anionic phosphate in DMEM and producing tiny crystallites. These crystallites gradually grew and became denser to form stable aPD‐L1@CaP nanoparticles. Subsequently, APCP was incorporated into the aPD‐L1@CaP nanoparticles through coordination interactions with Ca^2^⁺ ions. Analysis of the drug loading capacity revealed that the loading of aPD‐L1 and APCP increased proportionally with the dosage, achieving loading capacities of 15.7% for aPD‐L1 and 23.1% for APCP at a 1:3 drug loading ratio (**Figure** **2**A).

Dynamic light scattering (DLS) measurements indicated that the drug loading did not significantly affect the particle size, with the final aPD‐L1/APCP@CaP nanoparticles averaging approximately 170 nm in diameter (Figure 2B and Figure S1, Supporting Information). Transmission electron microscopy (TEM) revealed that the nanoparticles were spherical with a size of approximately 50 nm (Figure 2C). Elemental mapping analysis confirmed the composition of the nanoparticles, predominantly consisting of C, N, O, P, and Ca, indicating a stable calcium phosphate structure (Figure 2D). The aPD‐L1/APCP@CaP nanoparticles demonstrated excellent long‐term stability in aqueous medium, PBS buffer, and DMEM complete culture medium containing FBS (Figure S2, Supporting Information).

To evaluate the in vitro release profile, we used ELISA and HPLC methods to quantify the concentrations of aPD‐L1 and APCP, respectively. At pH 7.4, the release of aPD‐L1 and APCP was less than 20% within 24 h, suggesting effective encapsulation and prevention of premature drug release (Figure 2E). However, under simulated tumor microenvironment conditions (pH 6.4), the release exceeded 60% within 1 h, indicating pH‐responsive release characteristics. Thus, the nanoparticles stably encapsulate aPD‐L1 under normal physiological conditions, preventing interaction with normal tissues, while in the acidic tumor microenvironment, aPD‐L1 is rapidly released, restoring its therapeutic activity. TEM analysis of the nanoparticles under different pH conditions showed that they maintained their structure at pH 7.4 for up to 12 h, but rapidly disintegrated at pH 6.4 within 1 h (Figure 2F), demonstrating acid‐sensitive degradation. The hemolysis test showed that aPD‐L1/APCP@CaP did not damage mouse red blood cells, even at a concentration of 200 µg mL^−1^ (Figure S3, Supporting Information), indicating its hemocompatibility and suitability for intravenous administration. Additionally, the nanoparticles did not affect common clotting indicators in plasma, as shown by the thrombin time (TT), prothrombin time (PT), and activated partial thromboplastin time (APTT) measurements (Figure S4, Supporting Information), suggesting minimal thrombosis risks following administration.

For in vivo delivery evaluation, we labeled aPD‐L1 with Cy5.5 fluorescein and encapsulated it in the nanoparticles to create Cy5.5‐aPD‐L1/APCP@CaP. Following intravenous injection into B16F10 melanoma‐bearing mice, fluorescence imaging showed that Cy5.5‐aPD‐L1/APCP@CaP nanoparticles accumulated efficiently at the tumor site via the enhanced permeability and retention (EPR) effect, with maximum fluorescence intensity observed at 12 h post‐injection (Figure 2G, Figure S5, Supporting Information). These nanoparticles exhibited significantly higher tumor accumulation, with a 6.96‐fold increase in tumor localization compared to free Cy5.5‐aPD‐L1. After 24 h, the mice were euthanized, and the tumor tissue was analyzed using fluorescence microscopy. The Cy5.5‐aPD‐L1/APCP@CaP group showed strong Cy5.5 fluorescence distributed throughout the tumor tissue, in contrast to the free Cy5.5‐aPD‐L1 group, where fluorescence was weaker and localized to the tumor periphery (Figure 2H). Moreover, analysis of major organs at various time points revealed that Cy5.5‐aPD‐L1/APCP@CaP nanoparticles were mainly metabolized by the liver (Figure S6, Supporting Information). This indicates that nanoparticle delivery enhances both the accumulation and deep penetration of aPD‐L1 in tumor tissue, potentially improving immunotherapeutic efficacy while minimizing systemic toxicity. These findings underscore the potential of aPD‐L1/APCP@CaP nanoparticles for enhancing the therapeutic index of aPD‐L1 in melanoma treatment.

### 2.3 Enhanced Anti‐Tumor Efficacy of aPD‐L1 Delivered by CaP Nanoparticles In Vivo

**Figure 3:**
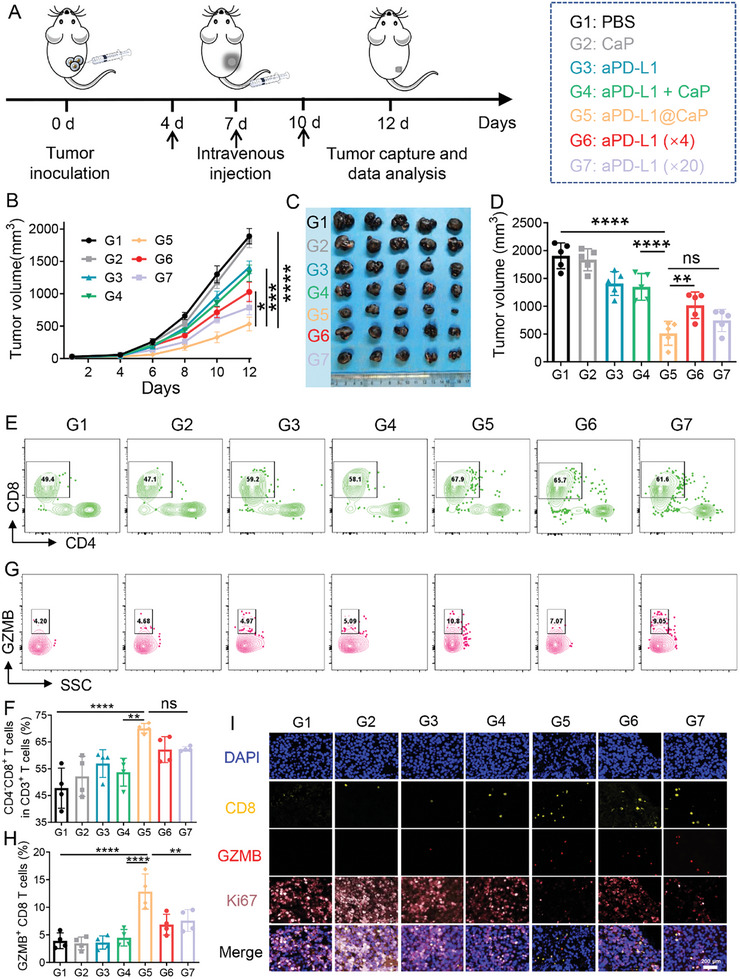
A) Schematic representation of treatments administered to mice with subcutaneous B16F10 tumors. Various formulations were intravenously injected on days 4, 7 and 10. B) Tumor growth curves of mice receiving different treatments. C) Photographs of tumors post‐treatment. D) Tumor weights of treated mice (*n* = 5). E) Flow cytometry plots and F) statistical analysis of the proportions of CD3⁺CD8⁺ T cells in B16F10 tumors. G) Flow cytometry plots and H) statistical analysis of GZMB⁺CD8⁺ T cells in B16F10 tumors (*n* = 4). I) Immunofluorescence staining of CD8, GZMB, and Ki67 expression in tumor tissues following different treatments. *p < 0.05, **p < 0.01, ***p < 0.001, ****p < 0.0001.

To investigate the enhancement of the anti‐tumor effect of aPD‐L1 via nanoparticle delivery, we prepared aPD‐L1@CaP nanoparticles loaded with aPD‐L1. After establishing the B16F10 tumor model, various treatments were administered, including PBS, CaP, aPD‐L1 (2.25 mg kg^−1^), aPD‐L1+CaP, aPD‐L1@CaP, aPD‐L1 (fourfold dose, 9 mg kg^−1^), and aPD‐L1 (20‐fold dose, 45 mg kg^−1^). These treatments were injected on the 4th 7th and 10th days, and tumor volume was continuously monitored (**Figure** **3**A). The efficacy was evaluated through tumor growth curves (Figure 3B), tumor photographs post‐treatment (Figure 3C), and tumor volume measurements (Figure 3D). The results demonstrated that free aPD‐L1 exhibited dose‐dependent anti‐tumor activity. Blank CaP showed no therapeutic effect, but when physically mixed with aPD‐L1, the efficacy was comparable to that of free aPD‐L1. The aPD‐L1@CaP group exhibited the most significant therapeutic effect, comparable to even the 20‐fold dose of free aPD‐L1. These results indicate that nanoparticle delivery enhances the efficacy of aPD‐L1, primarily due to improved accumulation in the tumor region. Further analysis using immune flow cytometry revealed that the aPD‐L1@CaP treatment group had increased infiltration of CD8⁺ cytotoxic T cells (Figure 3E,F, Figure S7, Supporting Information) and greater release of granzyme B (GZMB) (Figure 3G,H) in tumor tissues. Immunofluorescence staining showed that as cytotoxic T cells released GZMB, tumor cell proliferation in the aPD‐L1@CaP group was significantly inhibited (Figure 3I, Figure S8, Supporting Information). These findings suggest that delivering aPD‐L1 through CaP nanoparticles can significantly reduce the required dosage, achieving more efficient anti‐tumor immunotherapy.

### 2.4 In Vivo Detoxification of aPD‐L1 Delivered by CaP Nanoparticles

**Figure 4:**
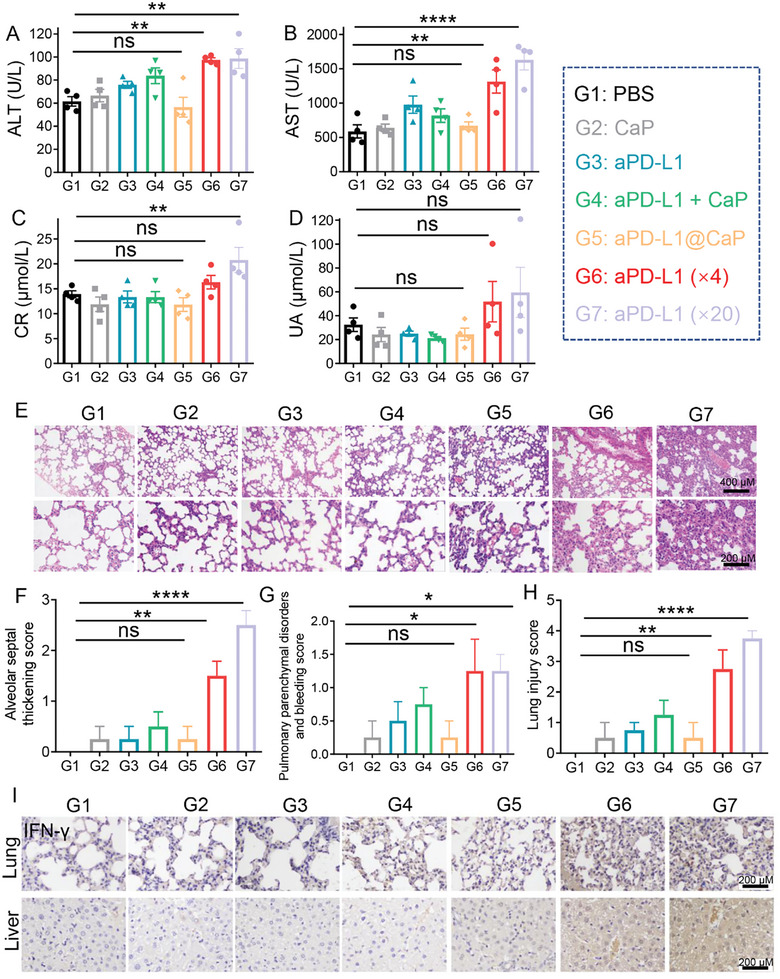
A) Serum levels of ALT and B) AST, and C) CR and D) UA after various treatments (*n* = 4). E) Representative H&E‐stained lung sections for lung injury. Scale bar: 400 µm. F) Alveolar septal thickening scores, G) pulmonary parenchymal disorders and bleeding scores, and H) total lung injury scores after various treatments (*n* = 4). I) IFN‐γ immunohistochemical staining of lung and liver tissues from mice receiving different treatments. Scale bar: 100 µm. **p < 0.01, ***p < 0.001, ****p < 0.0001.

Having demonstrated the enhanced in vivo efficacy of aPD‐L1 when delivered via CaP nanoparticles, we further investigated its potential for reducing the toxicity associated with aPD‐L1. ICIs, such as PD‐1/PD‐L1 inhibitors, can induce irAEs in various organs, including the skin, intestine, endocrine glands, liver, lungs, and kidneys,^[^ ^15^ ^]^ thus limiting their clinical applications. In comparison to PBS, intravenous injection of free aPD‐L1 resulted in dose‐dependent hepatorenal toxicity, evidenced by increased blood biochemical indicators such as AST/ALT and CR/UA (**Figure** **4**A–D). Elevated ALT levels are particularly noteworthy as they are considered more specific markers of liver injury than AST.^[^ ^16^ ^]^ The renal function damage induced by aPD‐L1 treatment manifested as acute kidney injury, reflected by elevated CR levels.^[^ ^17^ ^]^ Our study found that systemic injection of free aPD‐L1 led to varying degrees of CR and UA elevation. Conversely, treatment with aPD‐L1@CaP did not significantly alter these indicators.

Histopathological analysis of lung injury scores showed that systemic injection of aPD‐L1 caused partial thickening of alveolar septa and pulmonary consolidation, effects that were absent in the aPD‐L1@CaP group (Figure 4E–H). Immunohistochemical staining of the lungs and liver revealed that aPD‐L1 treatment significantly increased IFN‐γ expression, indicative of a systemic inflammatory cytokine storm (Figure 4I, Figure S9, Supporting Information). This increase in IFN‐γ expression was not observed in the aPD‐L1@CaP group. Thus, delivering aPD‐L1 through CaP nanoparticles can mitigate the systemic toxic side effects typically associated with aPD‐L1, including liver and kidney toxicity, lung injury, and cytokine storm.

### 2.5 Synergistic Effect of APCP and aPD‐L1 Co‐Delivered by CaP Nanoparticles

**Figure 5:**
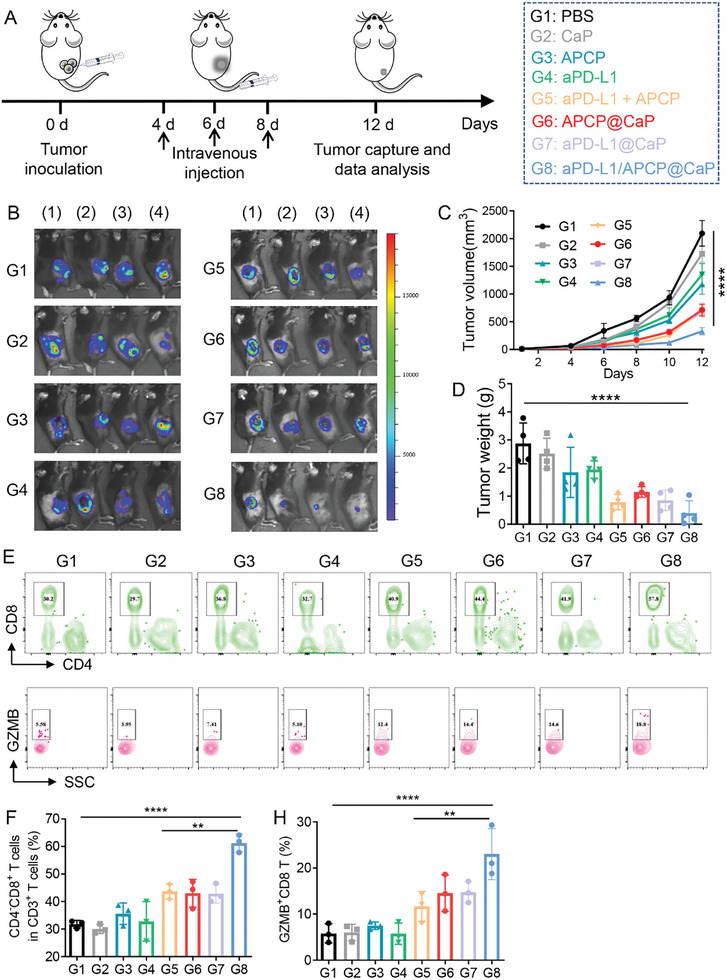
A) Schematic representation of treatments administered to mice with subcutaneous B16F10 tumors. PBS, CaP, APCP, aPD‐L1, APCP + aPD‐L1, APCP@CaP, aPD‐L1@CaP, and aPD‐L1/APCP@CaP were intravenously injected into B16‐F10 tumor‐bearing mice on days 4, 6, and 8. B) Whole‐body fluorescence imaging of tumor volume. C) Tumor growth curves. D) Tumor weights of tumor‐bearing mice receiving different treatments (*n* = 4). E) Flow cytometry plots and F) statistical analysis of the proportions of CD3⁺CD8⁺ T cells. G) Flow cytometry plots and H) statistical analysis of GZMB⁺CD8⁺ T cells in B16F10 tumors of mice receiving various treatments (*n* = 3). *p < 0.05, **p < 0.01, ***p < 0.001, ****p < 0.0001.

Following the demonstration of the enhanced efficacy and reduced toxicity of aPD‐L1 delivered by CaP nanoparticles, we evaluated the synergistic effect of APCP and aPD‐L1 co‐delivered by CaP. The LV‐12 (pGLVH6/luci05/Puro) plasmid was transfected into B16F10 cells to stably express luciferase, thereby creating spontaneous fluorescence tumor‐bearing mice. After successful modeling, the mice were randomly divided into eight groups and administered the following treatments: PBS, CaP, APCP, aPD‐L1, APCP+aPD‐L1, APCP@CaP, aPD‐L1@CaP, and aPD‐L1/APCP@CaP (**Figure** **5**A). Tumor volume was measured every two days, and D‐luciferin was injected on the 12th day to track the tumor in vivo.

The results indicated that both APCP and aPD‐L1 exhibited certain immunotherapeutic effects when used alone, but the effects were more pronounced when delivered through CaP, demonstrating the advantage of targeted delivery with nanocarriers (Figure 5B,C and Figure S10, Supporting Information). The combination of APCP and aPD‐L1 showed superior efficacy compared to single drug treatments, whether administered via free injection or CaP delivery, confirming the synergistic effect of the drug combination. Post‐treatment, tumor tissues were excised and weighed to directly assess the anti‐tumor effect, which aligned with fluorescence imaging and tumor volume measurements (Figure 5D). Further evaluation of the immunotherapy efficacy was conducted by measuring the infiltration of CD8⁺ T lymphocytes and the release of GZMB. Consistent with previous results, all treatment groups effectively activated the immune system, with the CD8⁺ T cells in the aPD‐L1/APCP@CaP group exhibiting the most significant activation and cytotoxic effects (Figure 5E–H, Figure S11, Supporting Information). It is well known that activated macrophages can be classified as pro‐inflammatory M1 or anti‐inflammatory M2 macrophages. M1 macrophages enhance tumor antigen presentation, thereby boosting the cytotoxic functions of other leukocytes like CD8^+^ T cells, whereas M2 macrophages act as immune suppressors in the tumor microenvironment.^[^ ^18^ ^]^ Our results indicate that the aPD‐L1/APCP@CaP treatment group shows an increase in M1 macrophages and a decrease in M2 macrophages (Figure S12A,B, Supporting Information). Regarding Treg cells, while the CD73 inhibitor reduces their proportion, the aPD‐L1/APCP@CaP treatment does not further decrease Treg cells compared to the APCP@CaP treatment alone (Figure S12C, Supporting Information). Moreover, the aPD‐L1/APCP@CaP treatment did not result in significant changes in NK cell proportions (Figure S12D, Supporting Information). Moreover, several critical immunocompetent cytokines in serum, such as IFN‐γ and TNF‐α, were measured to assess the systemic immune response after treatment of aPD‐L1/APCP@CaP nanoparticles. These cytokines could not only directly damage tumor cells but also activate antitumor immunity for enhanced efficacy. The results showed that aPD‐L1/APCP@CaP slightly increased IFN‐γ levels but had no impact on TNF‐α levels (Figure S13, Supporting Information), suggesting that aPD‐L1/APCP@CaP can moderately activate systemic immune responses to enhance anti‐tumor effects. To further investigate whether the immune activation induced by aPD‐L1/APCP@CaP leads to excessive immune response causing liver and kidney damage, we assessed liver and kidney function markers such as ALT and CR (Figure S14, Supporting Information). The results showed that aPD‐L1/APCP@CaP did not cause significant changes in these indicators.

### 2.6 aPD‐L1/APCP@CaP Prevents Tumor Recurrence through Immune Memory Effect

**Figure 6:**
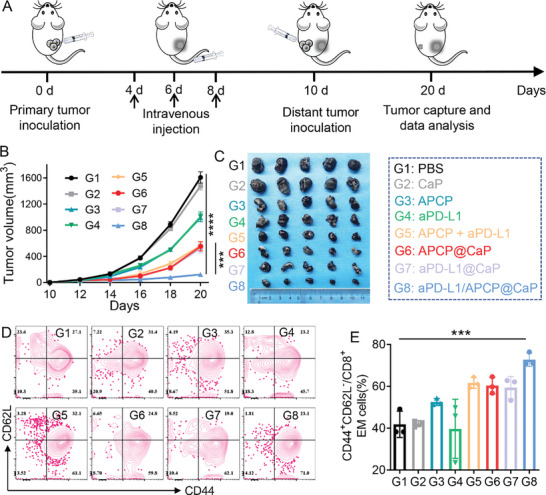
A) Schematic representation of the establishment of a bilateral B16F10 tumor model and the therapeutic regimen. PBS, CaP, APCP, aPD‐L1, APCP + aPD‐L1, APCP@CaP, aPD‐L1@CaP, and aPD‐L1/APCP@CaP were injected on days 4, 6, and 8 into mice with primary tumors on the right flank. A secondary distal tumor was inoculated on day 10, and distal tumor growth was monitored for 20 d. B) Distal tumor growth curves and C) tumor photographs of tumor‐bearing mice receiving various treatments (*n* = 5). D) Flow cytometric histograms and E) quantification showing the presence of TEM cells in distal tumors (*n* = 3). *p < 0.05, **p < 0.01, ***p < 0.001, ****p < 0.0001.

An important advantage of immunotherapy over traditional treatments is its ability to induce an immune memory effect, which helps inhibit tumor metastasis and recurrence.^[^ ^19^ ^]^ To investigate the potential of aPD‐L1/APCP@CaP nanoparticles in this regard, we examined their effect on the inhibition of distal tumor growth through the immune memory effect. Mice with primary tumors inoculated on the right flank were treated with PBS, CaP, APCP, aPD‐L1, APCP+aPD‐L1, APCP@CaP, aPD‐L1@CaP, and aPD‐L1/APCP@CaP on days 4, 6, and 8. On day 10, a secondary distal tumor was inoculated, and its growth was monitored for 20 d (**Figure** **6**A).

The therapeutic effects of each treatment on distal tumors mirrored their effects on primary tumors, with aPD‐L1/APCP@CaP demonstrating the most significant inhibition, achieving an inhibition rate of over 95% (Figure 6B,C). To elucidate the mechanism underlying the immune memory effect, we used flow cytometry to measure the levels of effector memory T cells (TEM) in distal tumors. The results showed a significant increase in TEM cells in distal tumors following treatment with aPD‐L1/APCP@CaP (Figure 6D,E and Figure S15, Supporting Information), confirming the ability of nanoparticles to activate long‐term immune memory and thereby prevent tumor metastasis and recurrence.

## 3 Conclusion

This study demonstrates the potential of CaP nanoparticles for the co‐delivery of aPD‐L1 and APCP, addressing the dual challenges of enhancing therapeutic efficacy and reducing toxicity in melanoma immunotherapy. The developed aPD‐L1/APCP@CaP nanoparticles showed excellent drug loading capacity, stability, and pH‐responsive release, ensuring targeted delivery to tumor tissues and minimizing premature drug leakage. In vivo experiments confirmed that the nanoparticle delivery system significantly improves aPD‐L1 accumulation in tumor regions, leading to enhanced anti‐tumor efficacy and reduced required dosages. The synergistic effect of APCP and aPD‐L1 was evident, further amplifying the therapeutic benefits. Importantly, this approach effectively mitigated systemic toxicity, preserving organ function and preventing immune‐mediated adverse events. The induction of an immune memory effect by the co‐delivery system highlights its potential to prevent tumor recurrence and metastasis, offering a durable and robust therapeutic option. This innovative strategy provides a new avenue for the formulation of combination therapies in melanoma and potentially other cancers, paving the way for more effective and safer immunotherapy treatments.

## 4 Experimental Section

### Materials

Calcium chloride dihydrate (CaCl_2_·2H_2_O) was obtained from Aladdin (Shanghai, China). Bovine serum albumin (BSA) was provided by Beyotime (Shanghai, China). The aPD‐L1 was purchased from Bioxcell (West Lebanon, USA). APCP was obtained from Tocris Bioscience (Bristol, UK). Sugar‐free DMEM medium was purchased from Solarbio (Beijing, China). Mouse IgG total ELISA kit was obtained from CUSABIO (Wuhan, China). Cyanine5.5‐aPD‐L1 (Cy5.5‐aPD‐L1) was purchased from Ruixi Biological Technology (Xian, China). The monoclonal antibodies, including IFN‐γ, CD8, GZMB, Ki67 used for immunofluorescence and immunohistochemical staining were purchased from ABclonal (Boston, USA). The antibodies used in flow cytometry analysis, including CD3, CD8, GZMB, CD44, CD62L were provided by Biolegend (San Diego, USA).

### Bioinformatics Analysis

The Xiangya Immunotherapy Cohort comprises 78 patients. Melanoma tissues were collected, and written informed consent was obtained from all patients. The inclusion of melanoma tissues was approved by the Ethics Committee of Xiangya Hospital, Central South University. The public melanoma immunotherapy transcriptome data was downloaded from the European Nucleotide Archive (ENA) (https://www.ebi.ac.uk/ena, PRJEB23709). Spatial transcriptomic data for this study were obtained from https://www.10xgenomics.com/cn/resources/datasets/human‐melanoma‐if‐stained‐ffpe‐2‐standard.

The correlation between CD73 and immune checkpoint expression was calculated using Spearman correlation, with significance considered if *p* < 0.05, indicating a significant correlation between CD73 and immune checkpoint expression. To determine the optimal cutoff point for CD73 expression in each cohort with respect to patients' overall survival (OS) and progression‐free survival (PFS), we employed the Maxstat software package. Based on the maximum selected log‐rank statistic, patients were stratified into high CD73 expression group and low CD73 expression group. Subsequently, the significance of differences using the log‐rank test was assessed.

### Preparation of aPD‐L1/APCP@CaP

Briefly, varying volumes (50, 100, 250, 500 µL) of aPD‐L1 (5 mg mL^−1^) were introduced into 10 mL BSA/sugar‐free DMEM solution (100 mg of BSA), and placed in the incubator for 2 h. Following this, 100 µL CaCl_2_ (1 m) was added and further incubated for 24 h. The aPD‐L1@CaP nanoparticles were obtained through centrifugation at 16 000 rpm for 15 min. Subsequently, different volumes (25, 50, 125, 250 µL) of APCP (10 mg mL^−1^) were added to the aPD‐L1@CaP and stirred for 12 h. The aPD‐L1/APCP@CaP nanoparticles were then collected by centrifugation with deionized water washing. Finally, all nanoparticles were freeze‐dried for subsequent use.

### Characterization of aPD‐L1/APCP@CaP

The particle size, polydispersity index (PDI), and ζ‐potential of aPD‐L1/APCP@CaP were detected using the Malvern Zeta Sizer Nano series (Zetasizer Nano ZS90, Malvern, UK). Morphological characteristics and elemental composition were confirmed through transmission electron microscopy (TEM, Tecnai G2 F20, FEI, US). The loading capacity (LC%) of APCP and aPD‐L1 were determined via high‐performance liquid chromatography (HPLC, Agilent 1260, USA) and mouse IgG total ELISA kit, respectively.

### In Vitro Drug Release Behavior

The 1 mg aPD‐L1/APCP@CaP was added into 10 mL PBS (pH 7.4 or 6.4, 10 mm) in a tube, which was placed in a shaking bed at a stirring speed of 100 rpm at 37 °C. At predetermined time intervals (1, 3, 6, 12, 24 h), 1 mL samples were withdrawn from the tube and centrifuged for 15 min at 16 000 rpm. HPLC and mouse IgG total ELISA kit was conducted to measure the concentration of APCP and aPD‐L1 in the supernatant, respectively.

### Hemolysis Assay

Fresh mouse blood was collected, and red blood cells were separated through multiple rounds of centrifugation and washing. These cells were then diluted in PBS to create a 2% suspension, which was incubated at 37 °C for 3 h with an equal volume of either water, PBS (10 mm), or Cy5.5‐aPD‐L1/APCP@CaP (200 µg mL^−1^). After incubation and centrifugation, the absorbance of the resulting supernatants was measured at 541 nm using a UV/vis spectrophotometer.

### Blood Coagulation Assay

Platelet‐poor plasma was prepared by centrifuging fresh citrate‐anticoagulated blood samples. This plasma was then mixed with various concentrations of aPD‐L1/APCP@CaP. Following incubation at 37 °C, the plasma supernatant was collected and analyzed for APTT, PT, and TT using an automated coagulation analyzer (Siemens Healthcare Diagnostics, Germany).

### In Vivo Biodistribution

Free Cy5.5‐aPD‐L1 or Cy5.5‐aPD‐L1/APCP@CaP (Cy5.5‐aPD‐L1: 2 mg kg^−1^) was intravenously injected into B16F10 tumor‐bearing mice, and imaged at 1, 2, 4, 8, 12 and 24 h by the IVIS system (Waltham, PerkinElmer, MA). After the images were acquired, the fluorescence intensity in the tumor sites was calculated. After 24 h, the mice were sacrificed, and tumor frozen sections were stained by DAPI and observed by confocal laser scanning microscopy (CLSM, ZEISS, Germany) to determine the tumor distribution of Cy5.5‐aPD‐L1. Moreover, five mice were sacrificed at 1, 2, 4, 8, 24 and 48 h after intravenously injection. The major organs, including heart, liver, spleen, lung and kidney were collected, and the fluorescence intensity was quantified using IVIS system.

### Evaluation of In Vivo Anticancer Immune Response and Adverse Effects of aPD‐L1 @CaP

A total of 5×10^5 B16F10 cells were subcutaneously injected into the right flank of 6 week old C57BL/6 mice. Then, when tumor volume was reached to 50 mm^3^, tumor‐bearing B16F10 mice were randomly allocated into seven groups. Each group received intravenous injections of various formulations: PBS, CaP, aPD‐L1 (2.25 mg kg^−1^), aPD‐L1 + CaP, aPD‐L1@CaP, aPD‐L1 (×4, 9 mg kg^−1^), and aPD‐L1 (×20, 45 mg kg^−1^). Tumor size and mouse weights were monitored every two days throughout the treatment period. Following treatment completion, tumors and major organs were collected and fixed for subsequent histopathological analysis. Additionally, blood serum was isolated to assess biochemical markers.

### Multiplex Immunohistochemical (Multi‐IHC) Assay

Tumor tissue slides (3–5 µm) were dewaxed and rehydrated using a gradient alcohol series, followed by antigen retrieval with citrate buffer (Servicebio, pH 6.0). For the multi‐IHC procedure, protein blocking was performed using a blocking dilution (PerkinElmer). The slides were then incubated with primary antibodies at room temperature for 1 h: anti‐CD8 (1:1000, Abcam, ab209775), anti‐GZMB (1:2000, Abcam, ab255598), and anti‐Ki67 (1:200, Abcam, ab16667). Following incubation, the secondary antibody (Opal Polymer HRP Ms+Rb, PerkinElmer) was applied for 10 min at room temperature. Tyramide signal amplification (TSA) visualization was carried out using the Opal Seven‐Color IHC Kit (PerkinElmer, NEL801001KT), according to the manufacturer's instructions. Afterward, the antibody‐TSA complex was removed using citrate buffer and microwave treatment. The staining procedure was repeated for multiple rounds of dyeing. Multi‐IHC of tumor tissue was performed in the following order: CD8 (Opal 690), GZMB (Opal 520), and Ki67 (Opal 570). Finally, the slides were counterstained with DAPI and mounted.

### Lung Injury Assessment

The right lung underwent fixation in 4% paraformaldehyde, followed by paraffin embedding and sectioning into 4–5 µm slices. Subsequently, these slices were stained using hematoxylin and eosin (H&E) staining. Evaluation of lung sections was conducted based on four key criteria: alveolar congestion, hemorrhage, neutrophil infiltration or aggregation in the airspace or vessel wall, and the thickness of the alveolar wall/hyaline membrane. Two independent pathologists, blinded to the samples, graded the degree of morphological alterations on a scale from 0 to 4. The scoring system delineated as 0 for normal lungs, 1 for mild involvement (<25%), 2 for moderate involvement (25–50%), 3 for severe involvement (50–75%), and 4 for very severe involvement (>75%).

### Immunohistochemistry

The lung and liver tissue were made into sections. After undergoing dewaxing and gradient alcohol dehydration, antigen repair was conducted utilizing a microwave. The sections were allowed to cool to room temperature and then washed three times with PBS. An immunohistochemistry pen was used to encircle the sections, followed by blocking of endogenous peroxidase for 10 min. The primary antibody against IFN‐r (1:500, abclonal) was apply onto the slides and incubated overnight at 4 °C in a humidified chamber. The next day, the slides were incubated with the secondary antibody for 30 min and stained with DAB to observe the color development. Subsequently, slides were counterstained with hematoxylin. Finally, after a series of dehydration steps, the sections were mount with neutral resin and cover slips. The images were captured by a microscopy (Ts2R, Nikon).

### In Vivo Anti‐Tumor Assay

To assess the synergistic anti‐tumor effects of aPD‐L1/APCP@CaP, 5 × 10^5 B16F10 cells, transfected with the with the LV‐12 (pGLVH6/luci05/Puro) to enable stable luciferase expression, were subcutaneously implanted into the right flank of 6‐week‐old C57BL/6 mice. Six days post‐implantation, when the tumor volume reached 50–100 mm^3, the mice were randomly divided into six groups. Following the monitoring and calculation of tumor volume and mouse weight, the tumor‐bearing mice were administered 100 µL of various formulations via tail vein injection every 3 d. The formulations included PBS, CaP, APCP (3 mg kg^−1^), aPD‐L1 (2.25 mg kg^−1^), APCP + aPD‐L1, APCP@CaP (equivalent to 3 mg kg^−1^ APCP), aPD‐L1@CaP, and aPD‐L1/APCP@CaP. Tumor volumes and mouse weights were measured every 3 d using the formula (length × width^2 × 1/2). After four treatment cycles, the mice were intraperitoneal injected with D‐luciferin sodium salt (40901ES01, Yeasen). Later, the tumor tissues were collected from each mouse for further analysis.

### In Vivo Antitumor Immune Response

To evaluate the immune response induced by aPD‐L1@CaP and aPD‐L1/APCP@CaP in vivo, tumors were excised, dissociated, and filtered through 40 µm cell strainers (BD Falcon) to obtain single‐cell suspensions. Dead cells were identified using the Zombie Aqua Fixable Viability Kit (BioLegend), following the manufacturer's protocols. The cell suspensions were subsequently stained with antibodies targeting specific surface markers. For the aPD‐L1@CaP group, the following markers were used: APC/Cyanine7 anti‐mouse CD45, APC anti‐mouse CD3, PerCP/Cyanine5.5 anti‐mouse CD4, and PE/Cyanine7 anti‐mouse CD8a (all from BioLegend). For the aPD‐L1/APCP@CaP group, an expanded panel of markers was used, including APC/Cyanine7 anti‐mouse CD45, APC anti‐mouse CD3, PerCP/Cyanine5.5 anti‐mouse CD4, PE/Cyanine7 anti‐mouse CD8a, APC anti‐mouse CD25, Brilliant Violet 605 anti‐mouse NK‐1.1, PE anti‐mouse CD11b, PerCP/Cyanine5.5 anti‐mouse I‐A/I‐E, PE anti‐mouse CD206, APC anti‐mouse F4/80, Brilliant Violet 711 anti‐mouse/human CD44, and PE anti‐mouse CD62L (all from BioLegend). For intracellular or nuclear staining, cells were fixed and permeabilized using the Foxp3/Transcription Factor Staining Buffer Set (eBioscience). Intracellular markers included PE/Dazzle 594 anti‐human/mouse Granzyme B and FOXP3 Monoclonal Antibody (FJK‐16s, PE, eBioscience). Stained cells were analyzed using the DxP Athena flow cytometry system (Cytek, USA), and the data were processed using FlowJo Software (version 10.4).

### Elisa Assay

Mouse TNF‐α and IFN‐γ levels were measured using ELISA kits (Sangon Biotech). Serum samples were collected following the completion of treatment, and TNF‐α and IFN‐γ levels were quantified according to the manufacturer's instructions.

### Immune Memory Effects In Vivo

Besides, the immune memory effects of aPD‐L1/APCP@CaP in inhibiting distant tumor growth were evaluated. Primary tumor‐bearing mice received various treatments, including PBS, CaP, APCP, aPD‐L1, APCP + aPD‐L1, APCP@CaP, aPD‐L1@CaP, and aPD‐L1/APCP@CaP (aPD‐L1: 2.25 mg kg^−1^, APCP: 3 mg kg^−1^). Subsequently, mice were challenged with a distant B16F10 tumor at the left hind leg. The body weight and volume of the distant tumors were monitored every 2 d.

### Statistical Analysis

Data are presented as the mean ± SEM. Statistical analyses were conducted using GraphPad Prism software (version 8.01). Comparisons among multiple groups were performed using one‐way ANOVA, followed by Tukey's multiple comparisons test. *p* < 0.05 was considered significant.

## Conflict of Interest

The authors declare no conflict of interest.

## Supporting information



Supporting Information

## Data Availability

The data that support the findings of this study are available from the corresponding author upon reasonable request.
