# Supplementary material for: Co‐Delivery of aPD‐L1 and CD73 Inhibitor Using Calcium Phosphate Nanoparticles for Enhanced Melanoma Immunotherapy with Reduced Toxicity
Source: Adv Sci (Weinh). 2024 Dec 24;12(7):2410545. doi: 10.1002/advs.202410545 (PMC11831434; doi:10.1002/advs.202410545)
Supplement: Supplementary file 1 — Supporting Information [file ADVS-12-2410545-s001.docx]

**Supporting Information**

**Co-Delivery of aPD-L1 and CD73 Inhibitor Using Calcium Phosphate Nanoparticles for Enhanced Melanoma Immunotherapy with Reduced Toxicity**

Peng Liu^3, 7#^, Jia Guo^2, 5, 6#^, Zuozhong Xie^4#^, Yusheng Pan^1^, Benliang Wei^8^, Ying Peng^1^, Shuo Hu^3, 7^, Jinsong Ding^1^, Xiang Chen^2, 5, 6^*, Juan Su^2, 5, 6^*, Hong Liu^2, 5, 6^*, Wenhu Zhou^1^*

^1^. Xiangya School of Pharmaceutical Sciences, Central South University, Changsha, Hunan, 410013, China

^2^. Department of Dermatology, Xiangya Hospital, Central South University, Changsha, Hunan, 410008, China

^3^. Department of Nuclear Medicine, Xiangya Hospital, Central South University, No. 87 Xiangya Road, Changsha, Hunan, 410008, China

^4^. Department of Otorhinolaryngology Head and Neck Surgery, The Second Xiangya Hospital, Central South University, Changsha, Hunan, 410011, China

^5^. National Engineering Research Center of Personalized Diagnostic and Therapeutic Technology, Changsha, Hunan, 410008, China

^6^. Hunan Key Laboratory of Skin Cancer and Psoriasis, Changsha, Hunan, 410008, China

^7^. Key Laboratory of Biological Nanotechnology, NHC. No. 87 Xiangya Road, Changsha, Hunan, 410008, China

^8^. Big Data Institute, Central South University, Changsha, Hunan, 410083, China

[^#^] These authors contributed equally to this work.

Email: chenxiangck@126.com

sujuanderm@csu.edu.cn

hongliu1014@csu.edu.cn

zhouwenhuyaoji@163.com


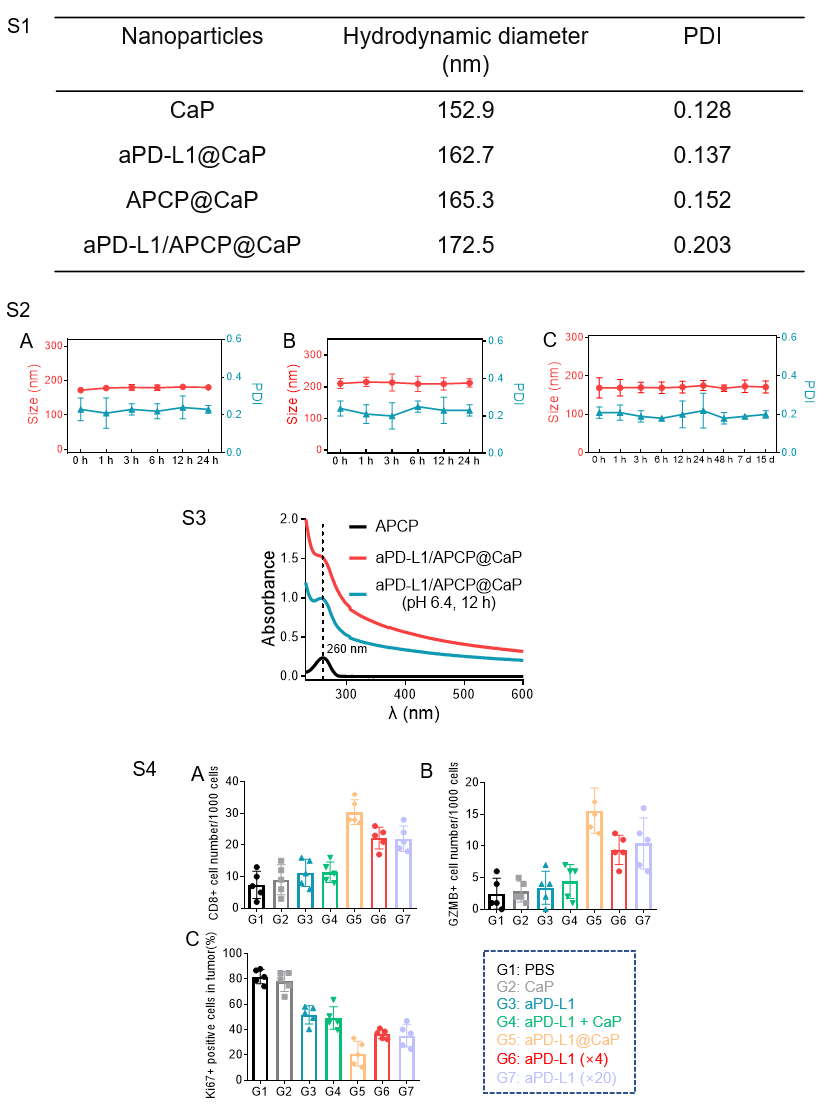


**Figure S1.** The hydrodynamic diameter and polydispersity index (PDI) of nanoparticles.


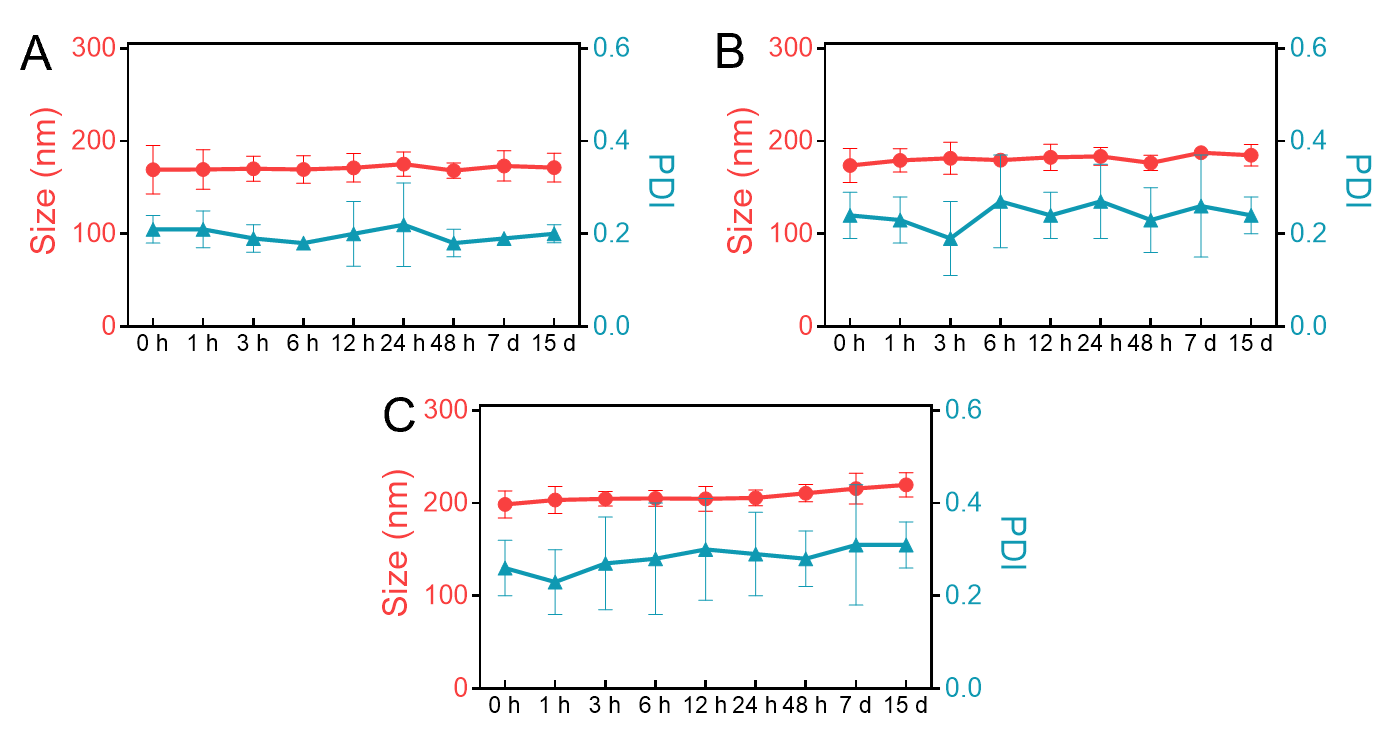


**Figure S2.** Dynamic monitoring the particle size of aPD-L1/APCP@CaP in (A) aqueous medium, (B) PBS buffer and (C) cell culturing medium containing 10% fetal bovine serum.


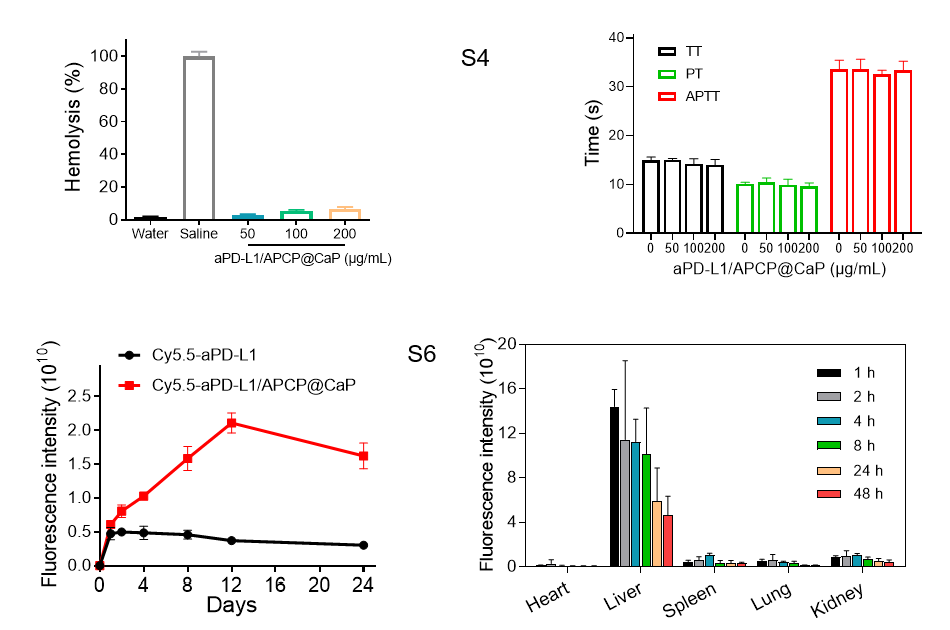


**Figure S3.** Hemolysis rate of red blood cells treated with various formulations.


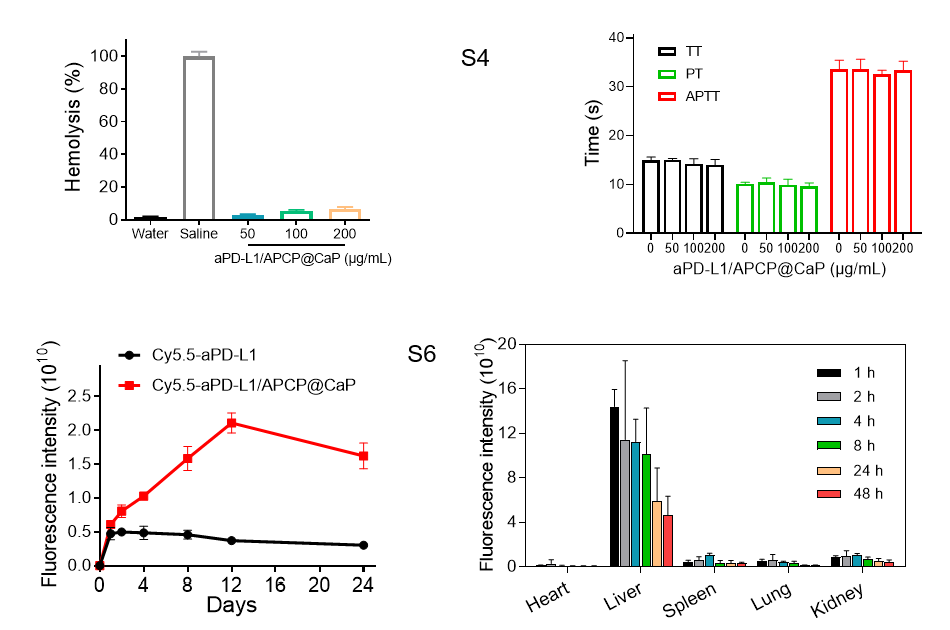


**Figure S4.** (A) TT values, (B) PT values and (C) APTT values indicated the coagulation function.


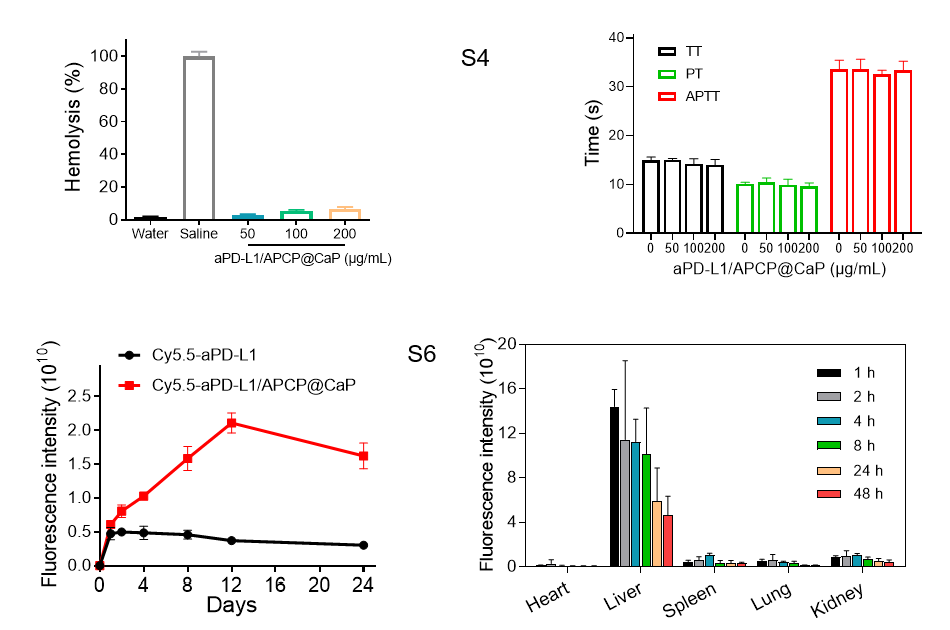


**Figure S5.** Fluorescence intensity of the tumor tissues after intravenous injection of Cy5.5-aPD-L1 or Cy5.5-aPD-L1/APCP@CaP at various time points.


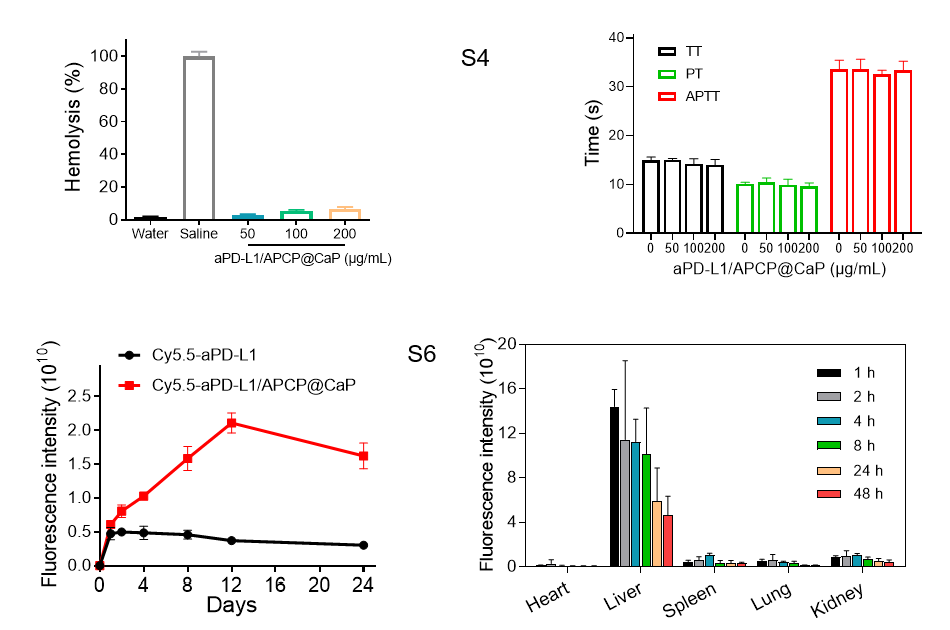


**Figure S6.** Quantitative analysis of fluorescence intensity of the major organs.


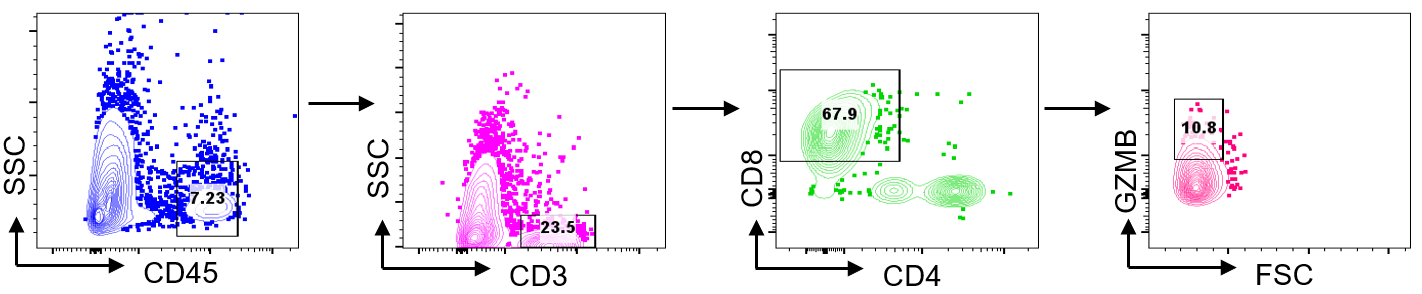


**Figure S7.** Flow cytometry gating strategy for the analysis of CD8^+^ T cells and GZMB in Figure 3.


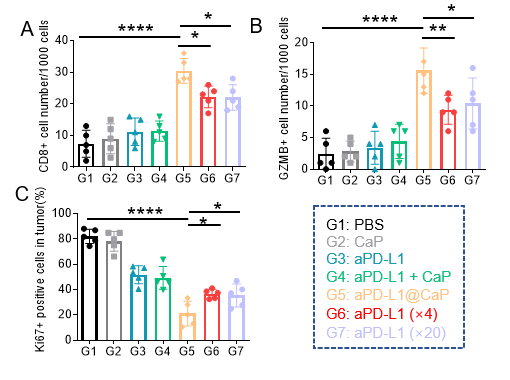


**Figure S8.** The quantitative analyses of CD8, GZMB and Ki67 expression in tumor tissues from immunofluorescence staining.


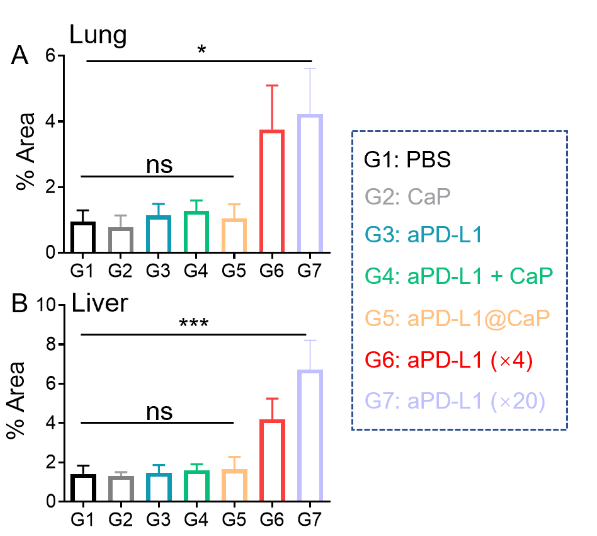


**Figure S9.** The quantification analysis of IFN-γ expression in lung and liver tissues from immunohistochemical staining.


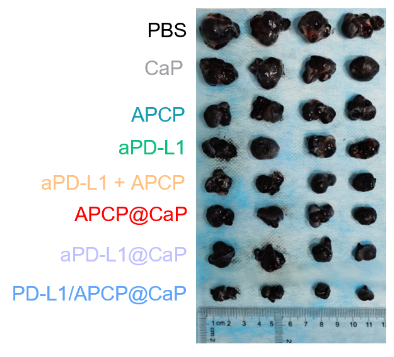


**Figure S10.** Photographs of tumors post-treatment in Figure 5.


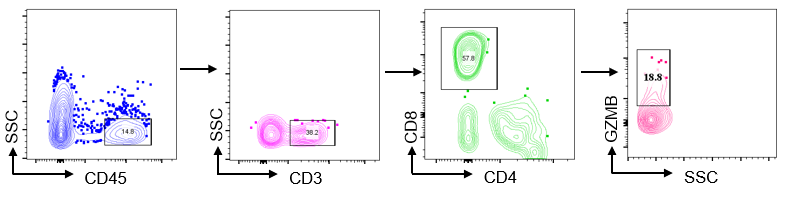


**Figure S11.** Flow cytometry gating strategy for the analysis of CD8^+^ T cells and GZMB in Figure 5.


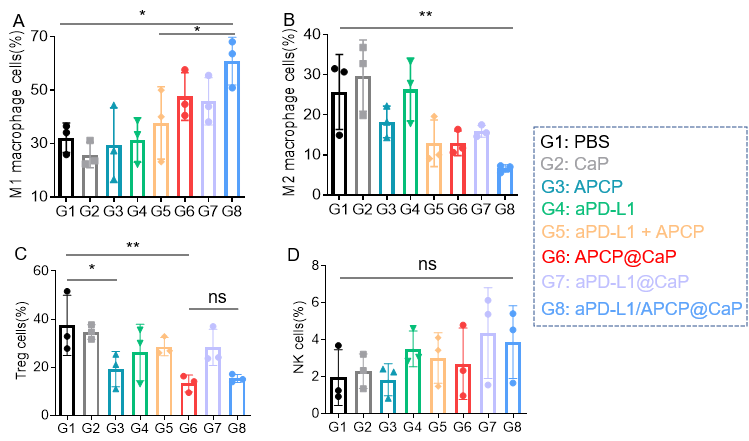


**Figure S12.** Statistical analysis of the proportions of (A) M1 macrophages, (B) M2 macrophages, (C) Treg cells and (D) NK cells in B16F10 tumors.


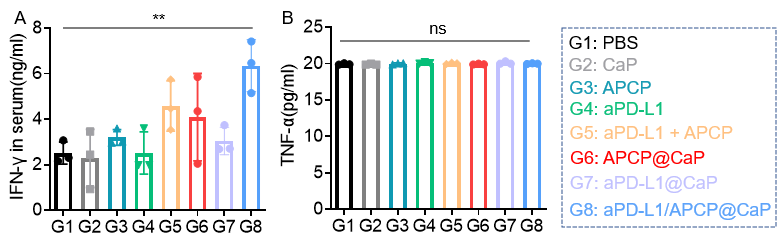


**Figure S13.** The secretion levels of (A) IFN-γ and (B) TNF-α in blood serum of mice


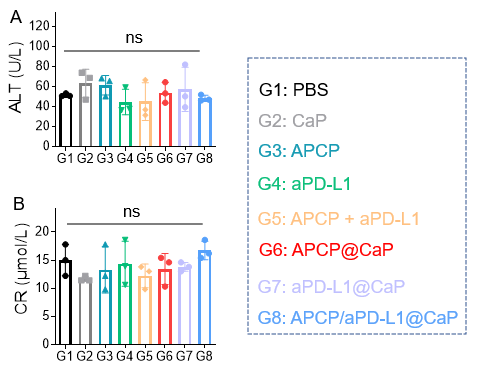


**Figure S14.** Serum levels of (A) ALT and (B) CR after various treatments


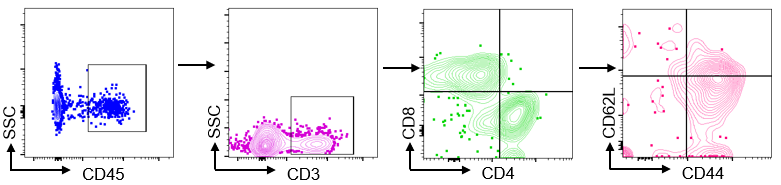


**Figure S15.** Flow cytometry gating strategy for the analysis of CD62L and CD44 cells in Figure 6.
